# 3DMGNet: 3D Model Generation Network Based on Multi-Modal Data Constraints and Multi-Level Feature Fusion

**DOI:** 10.3390/s20174875

**Published:** 2020-08-28

**Authors:** Ende Wang, Lei Xue, Yong Li, Zhenxin Zhang, Xukui Hou

**Affiliations:** 1 Shenyang Institute of Automation, Chinese Academy of Sciences, Shenyang 110016, China; ende_wang@sia.cn (E.W.); houxukui@sia.cn (X.H.); 2 Key Laboratory of Opto-Electronic Information Processing, Chinese Academy of Sciences, Shenyang 110016, China; 3 Key Laboratory of Image Understanding and Computer Vision, Liaoning Province, Shenyang 110016, China; 4 School of Information Science and Engineering, Shenyang Ligong University, Shenyang 110159, China; 1994512love@gmail.com; 5 College of Information Science and Engineering, Northeastern University, Shenyang 110819, China; 6 Key Lab of 3D Information Acquisition and Application, MOE, and College of Resource Environment and Tourism, Capital Normal University, Beijing 100048, China; zhangzhx@cnu.edu.cn

**Keywords:** deep learning, 3D model generation, multi-modal data constraints, feature fusion, attention mechanism

## Abstract

Due to the limitation of less information in a single image, it is very difficult to generate a high-precision 3D model based on the image. There are some problems in the generation of 3D voxel models, e.g., the information loss at the upper level of a network. To solve these problems, we design a 3D model generation network based on multi-modal data constraints and multi-level feature fusion, named as 3DMGNet. Moreover, 3DMGNet is trained by self-supervised method to achieve 3D voxel model generation from an image. The image feature extraction network (2DNet) and 3D feature extraction network (3D auxiliary network) are used to extract the features of the image and 3D voxel model. Then, feature fusion is used to integrate the low-level features into the high-level features in the 3D auxiliary network. To extract more effective features, each layer of the feature map in feature extraction network is processed by an attention network. Finally, the extracted features generate 3D models by a 3D deconvolution network. The feature extraction of 3D model and the generation of voxelization play an auxiliary role in the training of the whole network for the 3D model generation based on an image. Additionally, a multi-view contour constraint method is proposed, to enhance the effect of the 3D model generation. In the experiment, the ShapeNet dataset is adapted to prove the effect of the 3DMGNet, which verifies the robust performance of the proposed method.

## 1. Introduction

Generating the 3D digital model of object from an image by 3D reconstruction method is a challenging problem in the fields of computer vision and computer graphics. Rapid and automatic 3D model generation is the basis and an important part in the applications of digital city, virtual reality [1], and process simulation, etc. Particularly, the efficient generation of 3D models based on images can greatly reduce the workload of designers/users, and increase the real-time ability of human-computer interaction. Thus, it is of great significance for virtual reality and software design to quickly generate the corresponding 3D model according from an image captured by the mobile devices.

The core of 3D model generation is the discriminativeness of the extracted features, which determines the accuracy of 3D model generation. At present, deep learning-based 3D model generation has achieved robust performance, e.g., ShapeNet [2], PrGan [3], Pixel2mesh [4], ICV [5], PGGAN [6], and 3D-VAE [7]. These methods obtain a feature vector to represent the 3D model of the object through the coding of convolution network or manual design, and the feature vector generates a 3D model of the object. Many networks for 3D object generation models generally adapt 3D convolutions, composed of some extended 2D convolutions, to construct 3D feature extraction networks (e.g., 3DCNN [8]). For example, the TL-Net [9] leverages the 2DCNN and the 3DCNN to encode the image and 3D voxel model to a same latent space, respectively. Then the feature of the image is decoded to generate the 3D model. Although convolution neural network has made many excellent achievements in image processing, such as VGG [10], ResNet [11], and GoogleNet [12], the feature extraction of 3D model still faces some challenges. Recently, the commonly used 3D feature extraction network adopts coarse-to-fine feature extraction method in the process of the 3D model. The high-level network layer obtains highly abstract information, but loses the original details of the 3D model. Thus, the feature information becomes one of the factors, which affects the 3D model generation accuracy. To solve the above problems, Tchapmi et al. [13] propose the Segcloud network, in which the fine pixel set segmentation is obtained by adding a network of residual module. Inspired by the above idea, we propose a multi-level feature fusion method for robust 3D model feature extraction and representation. The backbone of our network is an extended version of autoencoder [14]. To make full use of the features in the lower and upper layers of the network, the feature extraction part of the network is improved by introducing a residual module. The residual module is the key design of the residual network [11]. In this research, the residual module is sandwiched between two convolutional operations. As a bridge between high-level features and low-level features, the information of the feature map near the input layer is integrated into subsequent high-level feature maps, to improve the robustness of the final 3D voxel model features. 

In order to reduce the redundancy and enhance the effectiveness of the feature generated by the feature fusion, we introduce the attention mechanism, based on the SeNet [15], which is a channel-wise attention method. For an image feature, the feature of each channel is multiplied by the weight to suppress the features of the unimportant channels and enhance the features of the important channels. Finally, the quality of features is improved. We apply this module to our 3DMGNet to deal with the features of each layer, which can strengthen the expression of effective feature information extracted by the 3D model and reduce the redundant feature information, to ultimately enhance the robustness of the 3D voxel model features.

Similar to the TL-Net [9], the proposed method adopts the idea of constraint modeling, which trains the network through data consistency constraints and feature constraints. The difference from the TL-Net is that, we improve the image feature extraction network and the 3D auxiliary network respectively, based on the premise of the constraint modeling idea that the 3D voxel features constrain the image features. Moreover, the loss function is also improved by introducing the way of multi-view contour constraints. 

**Figure 1:**
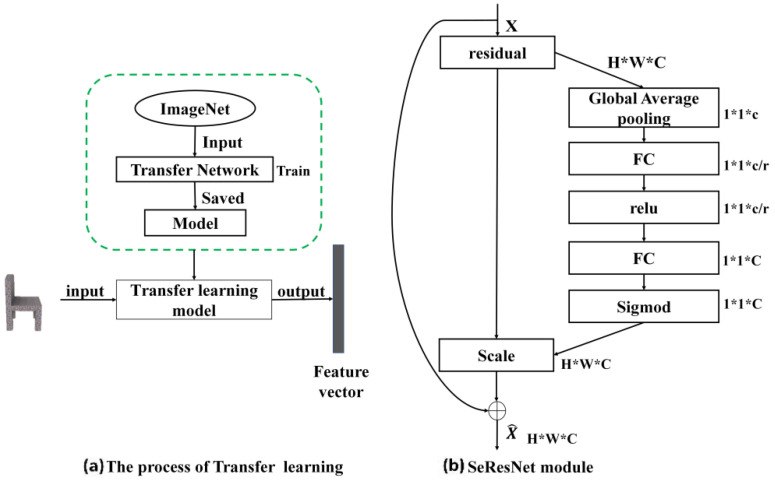
(**a**) The process of extracting 2D image features through transfer learning. (**b**) The basic module structure for Se-ResNet-101, which is used as our transfer learning model.

The main contributions of this research are as follows:

(1) An image feature extraction network is introduced in the 2DNet for the 3D model generation. As shown in Figure 1a, Se-ResNet-101 is implemented to extract image features based on transfer learning method. As shown in Figure 1b, SeNet [15] is embedded into the residual module of the ResNet [11], to construct the basic architecture of the Se-ResNet-101.
(2) A new 3D voxel model feature extraction network is proposed. For this network, the features of the high-level feature map and the low-level feature map are merged to enhance the quality and robustness of the 3D voxel features, by adding skip connections to the 3D Autoencoder structure. Besides, an attention mechanism is introduced to learn the weights of fusion features in each channel, and enhance the quality of the feature by feature redirection.
(3) We propose a novel 3D model generation network based on multi-modal data constraints and multi-level feature fusion, i.e., 3DMGNet. Notably, 3DMGNet combines a multi-view contour constraint into the construction of loss function, which improves the effect of the 3D models generation network.

## 2. Related Works

### 2.1. Volumetric 3D Modeling

Voxel is a kind of Euclidean structure data representation of 3D data [16]. Similar to the image, the 3D voxel model is composed of a regular grid, which is arranged in the 3D space. The existence or absence of the voxel grid in the 3D space is respectively represented by 1 or 0. Voxelization representation is widely used in 3D modeling tasks and other related 3D vision tasks. For example, VoxelNet [17], VConv-DAE [18], and LightNet [19,20,21].

The generation of 3D voxel models is one of the main research fields in 3D vision. The common characteristic of these networks is that the feature of the image is extracted by the network directly or indirectly, which is further used to reconstruct the 3D voxel model. The network involved in this process is also called encoder-decoder network.

Recently, to solve the problem of 3D voxel model generation, many researchers attempt to train a generation model by deep learning. For instance, Wu et al. [22] proposed the model of ShapeNet, which expresses 3D data in a voxelization form for the first time, and shows the presence or absence of each point in 3D space with a standard grid. The original 3D model of point cloud data or mesh data are easily processed with convolution network by voxelization representation. ShapeNet expands the 2D convolutional network to the 3D space, which provides inspiration for researchers to explore the various vision tasks, including the generation of 3D models. The 3DGAN [7] uses random noise to generate a 3D voxel model. The authors extend the 2D GAN (Generative Adversarial Network) structure to the 3D space. Furthermore, 3DGAN is composed of two parts: a generator network and a discriminator network. The generator network is used to generate a 3D model, and the discriminator network is adapted to determine the difference between the generated 3D model and the real 3D model. This network can generate a high-precision 3D model. However, the GAN is hard to train, which is a challenge for obtaining a robust model. Choy et al. [23] propose a 3D-R2N2 network, which designs the RNN (recurrent neural network) to learn the features of multiple images, to form the potential feature representation of the target object by the view of one or more objects. Lastly, a 3D object voxel model is generated. However, this method will not perform well when reconstructing models with poor texture. PrGAN [3] is proposed to obtain the 3D voxel model and viewpoint information of an object through a 3D up-sampling network with an initialized vector. The contour images are projected on the generated 3D model and the raw 3D model respectively. Finally, a generator is added to distinguish generation contour image from the real contour image. In fact, the authors adapt the idea of GAN [24] to improve the generation effect through data constraints, while Wu et al. [25] learn the geometric information and structural information of objects by two branches of RNN. Geometric information and structural information are composed of two parts: the voxel information of each part in 3D model, and the relationship between bounding boxes of the paired 3D voxel models. For this method, two kinds of information are merged to generate a higher-precision 3D model with more robust features. However, there is currently no guarantee that all generated shapes are of high quality.

### 2.2. Point Cloud Modeling

Compared with the voxel model, the unordered point cloud has the advantage of high storage efficiency, and can express the fine details of a 3D object, while the point cloud is not easily processed by a convolutional network. There has been much 3D model generation work based on the point cloud. For example, Mandikal et al. [26] propose 3DLMNet, in which a latent space is learnt by an auto-encoder network, and the image is encoded into the space. With the constraints of difference between the feature of the image and the feature of the point cloud, a space vector that can generate an image of a 3D model is obtained. Thus, the latent spatial representation is learnt in a probabilistic way, which can predict multiple 3D models in the image. Similar to the TL-Net [9], this network also adopts a multi-stage training method. Gadelha et al. [27] propose a tree-coding network to process point clouds, which can be combined with its tree-shaped decoding network to generate a 3D point cloud model. However, the proposed tree-coding network needs to represent the 3D model as a set of locality-preserving 1D ordered list. Jiang et al. [28] propose a conditional adversarial loss based single-view 3D modeling network, which is similar to the 3DGAN [7]. Besides, Li et al. [29] also propose the PC-GAN, which is directly extended from the GAN to the generation of 3D point clouds. Mandikal et al. [30] propose the Dense-PCR network, which is a deep pyramid network used for the point cloud reconstruction. A low-resolution point cloud algorithm is proposed, to achieve grid deformation by aggregating local and global point features, to increase the resolution of the grid hierarchically. However, certain predictions have artifacts consisting of a small cluster of points around some regions due to outlier points in the sparse point cloud, which obtains aggregated features in the dense reconstruction.

### 2.3. Mesh Modeling

The mesh model is a 3D model data representation composed of a series of vertices and triangular patches. Mesh model has its unique topology structure and cannot be easily processed by the convolutional network. Thus, mesh is first converted into spherical parameters or geometric images before the processing of the convolutional network. Some work related to the generation of mesh models are also proposed. For example, Sinha et al. [31] propose the SufNet, in which a geometric image of the mesh model is obtained through a spherical parametric method and corresponding information with the basic mesh, then a network generating the geometric image is designed. Because of the correlation between the geometric image and the raw mesh, the mesh model will be easily generated by the geometric image. However, SufNet can only reconstruct the surface of the rigid object. Pumarola et al. [32] propose a surface generation method of 3D model with geometric perception, which located the 2D mesh on the image through the 2D detection branch to detect the 2D position and confidence map of the mesh. Then, the vertices of each 2D mesh are raised to 3D through the 3D depth branch. The image clues are also used to further improve the quality of 2D detection. Then, the reconstruction branch generates the surface of the 3D model through the perspective projection method. This method can estimate the 3D shape of a non-rigid surface from a single image. Groueix et al. [33] propose AtalsNet inspired by the formal definition of the surface, which verifies that the surface part is similar to the topological space of the Euclidean plane. A shape embedded latent variable of the object is learnt by a method of square mapping to the local surface of the object. Finally, a mesh 3D model of the object is generated through a decoding network, but the AltasNet cannot capture the detailed information well.

## 3. The Proposed Method

**Figure 2:**
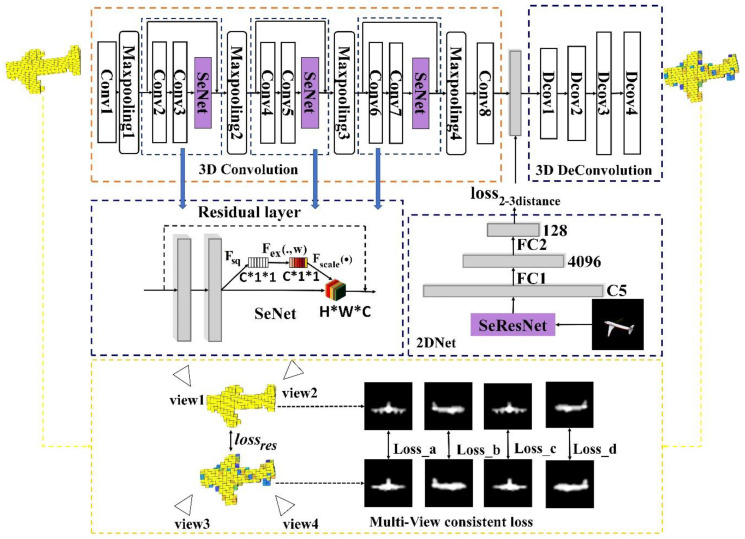
The proposed 3DMGNet network. In the 3D Convolution module, there are eight convolutional layers (Conv1-Conv8), four max-pooling layers (Maxpooling1-Maxpooling4), and three SeNet [15] module are separately connected after the Conv3, Conv4, and Conv5 layer. The specific structure of SeNet is shown in the middle left of the figure. In the 3D DeConvolution module, there are four Deconvolution layer-s (DeConv1-DeConv4). In the 2DNet module, the image feature is firstly extracted by Se-ResNet-101 [15] network, and then two fully connected layer changes the dimension of the feature to be as same as the dimension of the feature extracted by 3D Convolution module. The multi-view consistent loss is designed in the lower part of the figure to optimize the 3D auxiliary network.

Our network is composed of two parts: 3D auxiliary network and 3D model generation network. The 3D auxiliary network consists of two parts, i.e., 3D convolution and 3D deconvolution, as shown in Figure 2. The 3D model generation network consists of 2DNet and 3D deconvolution. The 2DNet is the image features extraction network. The 3D auxiliary network is responsible for obtaining a robust feature vector representation that represents the 3D model, by reconstructing the 3D model using the labels through the self-supervised learning way. The multi-level feature fusion of traditional 3D feature extraction is processed by 3D convolution. In order to improve the expression ability of the extracted features, we introduce an attention mechanism, which adds the SeNet into 3D convolution. The 3D model generation network is responsible for extracting the features of the image by using transfer learning. The image features are used to generate the 3D model of the object through the 3D up-sampling network (in the 3D auxiliary network). Similar to the GAN [23], the reconstruction accuracy of the 3D auxiliary network is controlled by reconstructing loss function $loss_{res}$. A constraint loss function $loss_{2-3distance}$ is constructed by comparing the L2 distance loss between the image feature in the 3D model generation network and the 3D model feature in the 3D auxiliary network. Additionally, four contour images of the generated voxel model and the original voxel model are selected to construct the multi-view contour loss $l_{contour}$, which designs view consistency constraints to improve the training effect and enhance the performance of the model generation network. Train in stages strategy is adopted to optimize the loss function designed in our network. Considering that the overall performance of the network is affected by the number of parameters and the amount of data, the 2D feature extraction network (2DNet) extracts the features of the image by designing transfer learning.

### 3.1. Image Feature Extraction

The Se-ResNet-101 network [15] is used in 2DNet to extract the image features. The Se-ResNet-101 is an extended version of ResNet [11], which has 101 layers. As shown in Figure 2, the output features of Se-ResNet-101-C5 layer is input as the feature map before the 2DNet. The 128-channel feature vector is obtained by the global average pooling and two fully connected operation, followed by the Se-ResNet-101-C5 layer. Here, we implemented transfer learning method, i.e., the weights trained on the ImageNet [34], are used as the pre-training model. The 128-channel feature vector is used to generate a 3D model through the 3D deconvolution part of the 3D auxiliary network.

### 3.2. Self-Supervised Learning and Multi-Level Feature Fusion Network for 3D Reconstruction

In order to solve the problem of the high-level features extracted from the 3D model lacking detailed information, the skip connections [13] are introduced to fuse the different levels of the feature, which simultaneously contain both local and global information of the 3D model.

**Figure 3:**
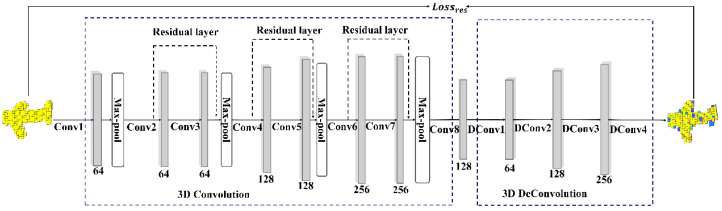
The 3D auxiliary network part of 3DMGNet.

As shown in Figure 3, the residual module is added in the 3D convolutional part of the 3D auxiliary network. Moreover, the 3D convolutional part consists of the convolution layers and the max-pooling layers. In the 3D convolution network, the three residual layers are designed. Furthermore, the 3D deconvolution network can extract a 128-dimensional feature vector of the 3D model. The 3D deconvolution part consists of Deconv1-Deconv4, which generates the 3D model from intermediate features.

For a network, the feature of the *i*-th layer is expressed as follow:

(1) $$F_{i}=Relu\left(\overset{N}{\underset{i=1}{\sum }}f_{i-1}\;*w_{i}+bias_{i}\right)$$

where $f_{i-1}$ is the feature of the (*i* − 1)-th layer, and N is the channel number of this feature map. *f*_0_ is the input data of the network. $w_{i}$ is the convolution kernel. $bias_{i}$ is the bias vector. The operator “*” represents the convolutional operation. Relu(·) is a nonlinear activation function.

The calculation process of 3D deconvolution is the inverse process of 3D convolution in the forward and back propagation process. The 3D deconvolution process is as follows:

(2) $$F_{i}^{\prime }=Relu\left(\overset{N}{\underset{i=1}{\sum }}w_{i}^{\prime }\;*\;f_{i-1}^{\prime }+bias_{i}\right)$$

where $f_{i-1}^{\prime }$ is the feature map of the (*i* − 1)-th layer, and N represents the channel number of the feature map. The kernel size of $w_{i}^{\prime }$ is *f_n_* × *f_n_* × *f_n_*. biasi is the bias vector. The $w_{i}^{\prime }$ in the deconvolution is the inverse matrix of the kernel in the convolutional calculation [35]. 

As shown in Figure 3, the residual layer is sandwiched between multiple convolution layers at different depth from the input layer, to blend the features of the layer closer to the input layer with the layers farther away. The calculation process is divided into a direct mapping part and a residual part. The output of a residual unit can be expressed as:

(3) $$H_{i}=\overset{N}{\underset{i=1}{\sum }}H_{i-1}+ℱ\left(H_{i-1}*W_{i-1}\right)$$

where $H_{i-1}$ represents the direct mapping part, which is the input of the residual layer. N represents the number of the input feature map. $ℱ\left(H_{i-1}*W_{i-1}\right)$ represents the residual part. $W_{i-1}$ is the kernel of each convolution layer in the residual part, which is composed of two convolutional operations.

### 3.3. The Attention Mechanism

**Figure 4:**
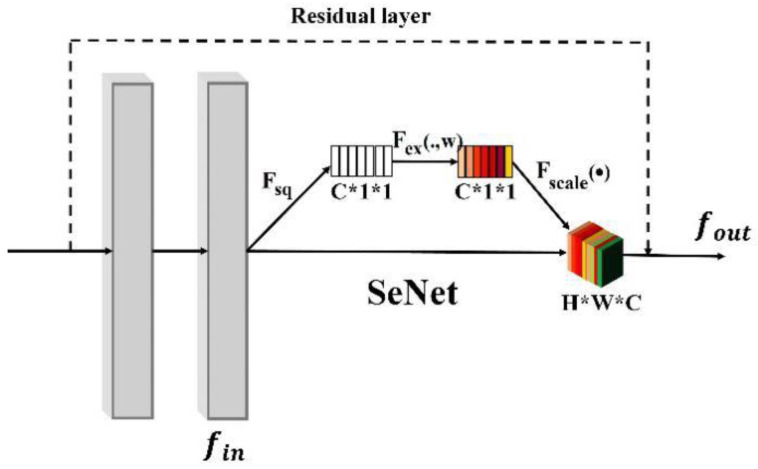
The attention mechanism based on SeNet [15]. The feature $f_{in}$ is firstly input the $F_{sq}$ module to obtain the global feature of each channel. Then, the feature is input to the $F_{scale}\left(*\right)$ module to calculate the weight value of each channel. Finally, the weight value of each channel is multiplied with the input feature $f_{in}$, and the feature is concatenated with the feature from the residual layer.

To make the extracted fusion features more representative, an attention module (i.e., SeNet [15]) is added to further process the fusion features. The weights of each channel feature are redirected to suppress the unimportant part of the feature and enhance the discriminative part. SeNet [15] is a channel attention mechanism, the output features are designed to pay more attention to the relationship among the channels of the learned features through the attention mechanism network structure. It also redefines the importance of each channel feature according to the learned weights. The attention mechanism usually can reduce the redundancy information to optimize the fusion features to obtain more robust features. As shown in Figure 4, SeNet includes three processes. $F_{sq}$ means obtaining the global feature of each channel. $F_{ex}\left(.,w\right)$ means learning the feature weight of each channel; $F_{scale}\left(*\right)$ means that redirecting to each channel to obtain the final optimized features with the learnt weights.

The designed network with the attention mechanism is shown in Figure 4, SeNet is added to the two-layer convolution of residual module. To learn the weights of the important channel features and suppress the feature information of the unimportant channels, the input feature is compressed by channels through weight redirection operations to obtain new weights of the model.

To obtain the global feature of each channel, the feature map obtained by the fusion of global features and local features is compressed for each channel through the max-pooling operation. The weight values of each channel are learnt by two fully connected layers. The feature calculation through SeNet is as follows:

(4) $$f_{out}=f_{in}*sigmoid(fc_{2}(fc_{1}\left(maxpool\left(f_{in}\right)\right))$$

where $f_{in}$ ais the input feature of SeNet module. maxpool() represents the global maximum pooling operation. $fc_{1}$ and $fc_{2}$ are fully connected operations. *Sigmoid*() is the activation function.

### 3.4. Multi-View Contour Constraints

To make the generated 3D model more efficient, the multi-view contour projection method is used to construct the constraint. In this paper, three contours are selected along the 45°, 90°, and 135° rotation angle around the model, and one contour in the case of an elevation angle of 90° and a rotation angle of 90°. These four contours can describe the outline of the object more effectively. Besides, these four contour maps can construct the corresponding multi-view map constraints, which generates a 3D model similar to the corresponding original 3D model.

The projection contour image is calculated by the following steps: (i) the rotation matrix rotmatrix(θ,γ) is obtained based on the parameters of the elevation angle θ and the rotation angle γ. (ii) The rotated 3D model $V_{\theta ,\gamma }\left(i,j,k\right)$ is obtained by coordinate transformation, which is performed on the coordinates of the 3D voxel model V. Each of its coordinate point c(i,j,k) is multiplied by the rotation matrix rotmatrix(θ,γ). (iii) Similar to the orthographic projection method, the formula $P=1-e^{-\underset{k}{\sum }V_{\theta ,\gamma }\left(i,j,k\right)}$ obtains the projection contour image of 3D voxel model, through cumulatively summing the number of voxels from the rotated angle under each light.

### 3.5. Loss Function

The loss function contains three parts: contour loss ($l_{contour}$), reconstruction loss ($loss_{res}$), two-dimensional and three-dimensional feature distance loss ($loss_{2-3distance}$).

(5) $$loss_{res}=-\overset{N}{\underset{i=0}{\sum }}\overset{N}{\underset{j=0}{\sum }}\overset{N}{\underset{k=0}{\sum }}p_{i,j,k}\log{\hat{p}}_{i,j,k}+\left(1-p_{i,j,k}\right)\log\left(1-{\hat{p}}_{i,j,k}\right)$$

(6) $$loss_{2-3distance}=\sqrt{\overset{n}{\underset{i=1}{\sum }}\left(z_{2d}-z_{3d}\right)}$$

(7) $$l_{contour}=\sqrt{\sum_{i=1}^{n}\left(r_{project}-pred_{project}\right)}$$

The output of 3DMGNet is the predicted probability value of every coordinate point in the voxel model. Cross-entropy calculates the loss function $loss_{res}$, where $p_{i,j,k}$ is the probability of a real voxel point, and ${\hat{p}}_{i,j,k}$ is the probability of the point that is predicted as a real voxel. The Euclidean distance is used in loss function $loss_{2-3distance}$ and $l_{contour}$ to calculate the distance between the features extracted by 2DNet and the features extracted by 3D auxiliary network, and the distance between the generated 3D model contour map and the original 3D model contour map. In $loss_{2-3distance}$ function, $z_{2d}$ and $z_{3d}$ represent the intermediate feature vectors extracted by image and 3D model, respectively. In $l_{contour}$ function, $r_{project}$ and $pred_{project}$ represent the projection of the real voxel model and the predicted 3D voxel model respectively.

After training, the reconstruction accuracy is promoted by the optimization of the loss function $loss_{res}$ and $loss_{contour}$. The distance between the features extracted from image and the features extracted from 3D model is minimized by optimizing the loss function $loss_{2-3distance}$. Thus, both of the 3D auxiliary network and 3D model generation network can produce the high accuracy of the 3D model. The overall loss function can be organized as follows:

(8) $$Loss_{total}=\lambda loss_{2-3distance}+\gamma loss_{res}+\mu loss_{contour}$$

where λ, γ, and u represent the proportion of $loss_{2-3distance}$, $loss_{res}$ and $loss_{contour}$ respectively.

### 3.6. The Training and Test of the Model

The training of 3DMGNet is divided into three stages. In the first stage, the 3D auxiliary network is trained, which uses reconstruction loss $loss_{res}$ and contour loss $loss_{contour}$ to guide the auxiliary network to reconstruct a high-precise 3D model. In the second stage, the parameter updating of the 3D convolution is firstly suppressed, then the loss function $loss_{2-3distance}$ constructs the feature of 2DNet and 3D convolution. In the third stage, the overall training of 3DMGNet network is accomplished, and the network stops training until each loss function is optimized.

We test the trained model through different datasets. The test network is composed of 2DNet and 3D deconvolution networks. The result of the test is voxel coordinate position and the probability that voxel coordinate point in the 3D model is judged as a real voxel point. By setting different thresholds, the coordinate points, whose probability value is lower than the threshold, are eliminated. Finally, we obtain the 3D model under different thresholds.

## 4. Results

In this section, we briefly introduce the experimental dataset, metrics, and implementation. Then, we describe the ablation study, experiment results analysis, and comparisons with other methods.

### 4.1. Dataset

The dataset that we used comes from ShapeNet [2]. Some classes in ShapeNet are chosen to build our experiment dataset, which includes plane, bench, chair, sofa, and monitor, etc. Table 1 shows the training and test data of each class. Each object datum is organized with two parts: the first part is the image of the object, which is obtained through 20 different views; the other part is the voxel model of the object.

### 4.2. Metrics and Implementation

#### 4.2.1. Metrics

Intersection over union (IOU) is used as the metric to evaluate the 3D model generation quality. IOU is the intersection over union between the generated voxel model and the real voxel model. For a generated voxel model I, 3D coordinate point of I is (x, y, z). Each coordinate point of the generated voxel model has its corresponding probability. Different thresholds have different IOU values.

(9) $$\mathrm{IOU}=\underset{i,j,k}{\sum }I({\hat{p}}_{i,j,k}>t)I\left(p_{i,j,k}\right)/\underset{i,j,k}{\sum }I\left(I\left({\hat{p}}_{i,j,k}>t\right)\;\mathrm{or}\;I\left(p_{i,j,k}\right)\right)$$

where $p_{i,j,k}$ and ${\hat{p}}_{i,j,k}$ represents the probability values of the predicted voxel and the real voxel respectively. I(·) presents the indicator function, and *t* represents the threshold value. The numerator means the number of intersection points, and the denominator means the number of union points.

#### 4.2.2. Implementation

In this research, the whole network is conducted in the way of phased training, and the loss function is optimized by the momentum optimizer. The learning rate in each stage is set as 0.0001, 0.0005, and 0.00005. The λ, γ and μ are set as 1.0, 0.9 and 0.8, respectively. The batch size is set as 10, the training epoch is set as 400. The input size of the image is 224 × 224 (the image is rendered by 3D model), and the resolution of the input and generated voxel model is 32 × 32 × 32.

We implemented experiments on a PC with an RTX2070 GPU.

### 4.3. Experiment and Comparison

#### 4.3.1. Ablation Studies and Comparisons

To adequately test the proposed 3DMGNet, we consider the following settings for the ablation experiment:

(a) TL-Net: The TL-Net [9] method is a single-view 3D model generation method based on voxel data. We directly use the original network as the baseline in the experiment.
(b) Voxel-Se-ResNet: This network combines the Se-ResNet-based transfer learning method with our baseline to evaluate the effectiveness of Se-ResNet-based transfer learning in the proposed 3D model generation.
(c) Voxel-ResidualNet: This network combines the multiple feature fusion and attention mechanism with the feature extraction of 3D voxel model in our baseline to evaluate the effectiveness of multi-level feature fusion and attention mechanism in our 3D model generation.
(d) 3DMGNet: The loss of multi-view contours is added to the Voxel-SeNet network, to verify the overall performance of the designed 3DMGNet.

To validate each module in the proposed method, we establish three groups of experiments to evaluate the effects of the 2DNet module, the multi-layer feature fusion module, and the multi-view contour constraints.

As shown in Table 2, there are three experiments, including Voxel-Se-ResNet-101, Voxel-ResidualNet, and 3DMGNet. They are trained and tested on six categories of object models, i.e., plane, bench, sofa, monitor, speaker and telephone. IOU values of the generated 3D models are obtained by each improved method when the threshold values are 0.1, 0.3, 0.5, 0.7, and 0.9 respectively.

**Figure 5:**
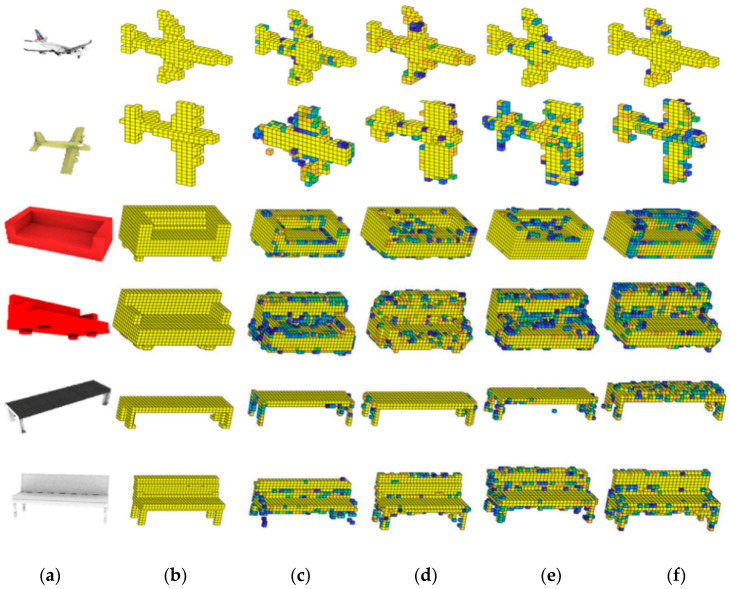
Visualization of model generation results of each category predicted by our improvement methods. (**a**) The original image, (**b**) The original voxelization of 3D model, (**c**) TL-Net [9], (**d**)Voxel-Se-ResNet-101, (**e**)Voxel- ResidualNet, **(f**) 3DMGNet. The probability value was stored in each voxel grid; blue represents the probability value is close to 0, yellow represents the probability value is close to 1.

To evaluate the effect of generating 3D models intuitively, the 3D models generated by different methods are visualized. As shown in Figure 5, the first two rows are different aircraft, the third and the fourth rows are different sofas, and the last two rows are different benches. In the generated 3D models, yellow point represents that there is a point with the predicted probability of 1, and different colors of points represent different predicted probabilities.

It can be seen in Figure 5 that the 3D models generated by our method are closer to the ground truth (Figure 5b), and the compared methods have generated many error points (e.g., the plane and bench in the second raw and sixth raw in Figure 5, respectively).

The results of ablation experiment on difference thresholds are shown in Table 3; we can obtain the following observations.

(a) The Voxel-Se-ResNet usually outperforms the baseline network TL-Net [9]. For plane, sofa, and bench, the IoU of Voxel-Se-ResNet-101 is at least 0.026 higher than TL-Net, as the improved 2DNet can extract better 2D image features through Se-ResNet-101, to promote the accuracy of the generated model.
(b) The Voxel-ResidualNet outperforms the baseline network (TL-Net [9]) and Voxel-Se-ResNet in most cases, as the more robust 3D model features are obtained through multi-level feature fusion and attention mechanism. The 3D model generation ability of 3D auxiliary network is improved, and the overall network performance is promoted to further constraint the image feature. Thus, the accuracy of the 3D model generation base on image is improved.
(c) The 3DMGNet outperforms TL-Net [9], Voxel-Se-ResNet, and Voxel-ResidualNet. The main reason is that the multi-view contour constraint is added to the 3DMGNet, which proves that the reconstruction accuracy of the 3D auxiliary network is improved.
(d) When the threshold is 0.1, 0.3, 0.5, and 0.7, the IOU value of 3DMGNet does not change much. For example, the difference in IOU values of plane, sofa, and bench at different thresholds is less than 0.011. When the threshold is set to 0.3, our method usually achieves the best performance.

However, 3DMGNet acquires similar accuracy in some categories to the Voxel-Se-ResNet-101 and Voxel-Residual, e.g., plane, which proves that the improved reconstruction performance of the auxiliary network has a better supervision effect on the performance of the final 3D model, although there is always a certain spatial position offset between the generated 3D model and the original 3D model, which will affect the accuracy of the 3D model generation.

#### 4.3.2. Comparison with Other Methods

In order to verify the effectiveness and advantages of the proposed 3DMGNet, we compare 3DMGNet with state-of-the-art methods, i.e., 3D-R2-N2 [23], OGN [36], DRC [37], Pix2Vox-F [38], Pix2Vox++/F [39]. In order to unify the comparison conditions, we follow the same experiment settings as in PixelVox [38], and compare the IOU results when the threshold is 0.3. The comparison results are shown in Table 4. In Table 4, the result values of compared methods come from Ref. [38,39].

The comparison results with other methods are shown in Table 4. From Table 4, we can obtain the following observations:

(1) The 3DMGNet outperforms 3D-R2-N2 [23], OGN [36], DRC [37]. Compared with 3D-R2-N2 [23], OGN [36] and DRC [37], 3DMGNet can achieve the best generation results, which obtains the highest IOU value, except for the category of Watercraft.
(2) The 3DMGNet can achieve better generation accuracy than PixVox-F [38] and PixVox++/F [39] in most categories, i.e., Airplane, Bench, Chair, Display, Lamp, Rifle, Sofa and Telephone. Pix2Vox-F and PixVox++/F solves the single-view-based 3D model generation problem by spatial mapping, while the 3DMGNet solves this problem from the perspective of multi-modal feature fusion. Although PixVox++/F can achieve the best average IOU, which is mainly caused by the fact that the IOU value of Speaker is obviously higher than 3DMGNet; the 3DMGNet performs its best performance in most categories of objects (at least nine categories).

In Table 4, although 3DMGNet has not achieved the best results in some categories, this does not affect the effectiveness of the proposed method. The 3D model generation accuracy is severely affected by the limited image information, which is the main challenge of single-view 3D model generation. The multi-modal feature constraint strategy is adopted in our method to solving this problem, but it is inevitable that the extracted features cannot be effective for all objects. Thus, the accuracy of 3DMGNet in some categories is slightly lower than the compared methods.

#### 4.3.3. Multi-Category Joint Training

To verify the generation ability of 3DMGNet, a multi-category joint training experiment is conducted to obtain the joint training model, i.e., Joint-3DMGNet. Multiple class models (plane, sofa, and bench) are randomly input to the 3DMGNet to train a model, which can generate multiple category 3D models based on image. The experiment results are compared with the original TL-Net [9], Diect-2D-3D (the direct trained model Diect-2D-3D, which consists only of the 2DNet and the 3D deconvolution part). The structure of the 3DMGNet and Joint-3DMGNet is completely the same. Considering the threshold values of 0.3, 0.5, and 0.7 can obtain better performance of 3DMGNet in Table 4, we compare the results when the threshold value is respectively set as 0.3, 0.5, and 0.7 in this experiment.

We have the following observations in Table 5:

(1) The Joint-3DMGNet outperforms the baseline network TL-Net [9], which proves that the combination of multi-modal feature fusion of 3D auxiliary network, multi-view contour constraint, and the improved image feature extraction network are effective for higher reconstruction performance.
(2) The IOU of Joint-3DMGNet is at least 0.023 higher than Direct-2D-3D, mainly because the image lacks spatial information, and it is not easy to generate a 3D model with higher accuracy without an auxiliary network part.
(3) For the multi-category joint training results, the best IOU results are achieved by Joint-3DMGNet in most cases, which illustrates that the Joint-3DMGNet can achieve better generalization performance than the compared methods.

## 5. Conclusions

We propose a 3D model generation method based on multi-modal data constraints and multi-level feature fusion. In this method, we design a self-supervised method to learn a robust 3D feature for the input voxel data. Specifically, the multi-level feature fusion is used to enhance the robustness of the extracted 3D features, and the attention mechanism is introduced to optimize the quality of the features. Therefore, the performance of the 3D model generation network is improved. To further improve the accuracy of the generated 3D model by the 3D auxiliary network, we also introduce a multi-view contour constraint to construct the constraint loss function. During the training stage, the similarity between the generated model and the original 3D model is constrained by the multi-view contour loss, which can effectively increase the accuracy of the generated 3D model.

In future, we would like to further explore the application of feature fusion and attention mechanisms in 3D model generation, 3D shape synthesis, and other tasks. Besides, 3D model generation from the geometric information and structural information, or global and local features of the 3D data, will be considered.

## Figures and Tables

**Table 1:** The number of each class in dataset.

Class	Total Number	Training Data	Test Data
Plane	3641	2831	810
Bench	1635	1271	364
Cabinet	1415	1100	315
Car	6748	5247	1501
Chair	6101	4744	1357
Monitor	986	766	220
Lamp	2087	1622	465
Speaker	1457	1132	325
Rifle	2135	1660	475
Sofa	2857	2222	635
Table	7659	5956	1703
Telephone	947	736	211
Watercraft	1746	1357	389

**Table 2:** NetWork structure comparison.

Name	3D Encoder	3D Decoder	2D Encoder
TL-Net	3DConv	Deconvolution	AlexNet
Voxel-SeResNe	3DConv	Deconvolution	Se-ResNet
Voxel-ResidualNet	3DConv+Residual+SeNet	Deconvolution	Se-ResNet
3DMGNet(Ours)	3DConv+Residual+SeNet+Mvcontour	Deconvolution	Se-ResNet

**Table 3:** The ablation study results (IoU) on difference thresholds. Note that the best results are highlighted in bold.

		Threshold				
		0.1	0.3	0.5	0.7	0.9
**Plane**	TL-Net	0.6565	0.6579	0.6545	0.6481	0.6315
	Voxel-Se-ResNet	**0.7041**	**0.7062**	0.7005	0.7022	0.6942
	Voxel-ResidualNet	0.6713	0.6839	0.6860	0.6837	0.6709
	3DMGNet(Ours)	0.6975	0.7039	**0.7047**	**0.7031**	**0.6948**
Sofa	TL-Net	0.6378	0.6405	0.6380	0.6319	0.6143
	Voxel-Se-ResNet	0.6683	0.6665	0.6639	0.6605	0.6523
	Voxel-ResidualNet	0.6800	0.6854	0.6830	0.6764	0.6560
	3DMGNet(Ours)	**0.6939**	**0.6973**	**0.6941**	**0.6867**	**0.6659**
Bench	TL-Net	0.4683	0.4589	0.4460	0.4277	0.3908
	Voxel-Se-ResNet	0.5364	0.5310	0.5253	0.5183	0.5035
	Voxel-ResidualNet	0.5503	0.5567	0.5492	0.5342	0.4937
	3DMGNet(Ours)	**0.5761**	**0.5803**	**0.5778**	**0.5711**	**0.5524**
Monitor	TL-Net	0.5052	0.4996	0.4935	0.4836	0.4625
	Voxel-Se-ResNet	0.5223	0.5131	0.5004	0.4829	0.4462
	Voxel-ResidualNet	0.5111	0.5103	0.5031	0.4835	0.4380
	3DMGNet(Ours)	**0.5335**	**0.5335**	**0.5189**	**0.4912**	**0.4250**
Speaker	TL-Net	0.6157	0.6105	0.6030	0.5911	0.5621
	Voxel-Se-ResNet	0.6150	0.6095	0.6039	**0.5969**	**0.5811**
	Voxel-ResidualNet	0.6164	0.6111	0.6028	0.5896	0.5579
	3DMGNet(Ours)	**0.6197**	**0.6175**	**0.6065**	0.5861	0.5367
Telephone	TL-Net	0.7649	0.7689	0.7697	0.7694	0.7654
	Voxel-Se-ResNet	0.7724	0.7748	0.7754	0.7754	0.7736
	Voxel-ResidualNet	0.7863	0.7907	**0.7900**	**0.7868**	**0.7744**
	3DMGNet(Ours)	**0.7875**	**0.7920**	0.7896	0.7828	0.7633
**Average**	TL-Net	0.6081	0.6061	0.6008	0.5920	0.5711
	Voxel-Se-ResNet	0.6364	0.6335	0.6282	0.6227	0.6085
	Voxel-ResidualNet	0.6359	0.6367	0.6357	0.6257	0.5985
	3DMGNet(Ours)	**0.6514**	**0.6541**	**0.6486**	**0.6368**	**0.6064**

**Table 4:** Comparison results (IOU) with other state-of-the-art methods. Note that the best results are highlighted in bold.

Class	3D-R2-N2 [23]	OGN [36]	DRC [37]	Pix2Vox-F [38]	Pix2Vox++/F [39]	3DMGNet
Airplane	0.513	0.587	0.571	0.600	0.607	**0.704**
Bench	0.421	0.481	0.453	0.538	0.544	**0.580**
Cabinet	0.716	0.729	0.635	0.765	**0.782**	0.741
Car	0.798	0.828	0.755	0.837	**0.841**	0.806
Chair	0.466	0.483	0.469	0.535	0.548	**0.566**
Display	0.468	0.502	0.419	0.511	0.529	**0.534**
Lamp	0.381	0.398	0.415	0.435	0.448	**0.455**
Speaker	0.662	0.637	0.609	0.707	**0.721**	0.618
Rifle	0.544	0.593	0.608	0.598	0.594	**0.628**
Sofa	0.628	0.646	0.606	0.687	0.696	**0.697**
Table	0.513	0.536	0.424	0.587	**0.609**	0.586
Telephone	0.661	0.702	0.413	0.770	0.782	**0.792**
Watercraft	0.513	**0.632**	0.556	0.582	0.583	0.600
**Average**	0.560	0.596	0.545	0.634	**0.645**	0.639

**Table 5:** The Joint-TRAINING results (IOU) on different thresholds. Note that the best results are highlighted in bold.

	Method	Threshold		
		0.3	0.5	0.7
**Plane**	TL-Net	0.6579	0.6545	0.6481
	Diect-2D-3D	0.6212	0.5914	0.5044
	Joint-3DMGNet	**0.6628**	**0.6642**	**0.6599**
Sofa	TL-Net	0.6405	0.6380	**0.6319**
	Diect-2D-3D	0.4228	0.3527	0.1699
	Joint-3DMGNet	**0.6576**	**0.6460**	0.6306
Bench	TL-Net	0.4589	0.4460	0.4277
	Diect-2D-3D	0.5429	0.5038	0.3559
	Joint-3DMGNet	**0.5452**	**0.5413**	**0.5281**
**Average**	TL-Net	0.5858	0.5795	0.5692
	Diect-2D-3D	0.5290	0.4826	0.3434
	Joint-3DMGNet	**0.6219**	**0.6172**	**0.6062**
